# Effect of hip joint angle at seat-off on hip joint contact force during sit-to-stand movement: a computer simulation study

**DOI:** 10.1186/s12938-018-0610-5

**Published:** 2018-11-29

**Authors:** Takuma Inai, Tomoya Takabayashi, Mutsuaki Edama, Masayoshi Kubo

**Affiliations:** 1 Institute for Human Movement and Medical Sciences, Niigata University of Health and Welfare, 1398 Shimami-cho, Kita-ku, Niigata City, Niigata 950-3198 Japan; 2 Department of Rehabilitation, Oguma Orthopedics Clinic, 5-8-9 Koshin, Nishi-ku, Niigata City, Niigata 950-2023 Japan

**Keywords:** Sit-to-stand movement, Hip joint contact force, Hip joint angle, Seat-off

## Abstract

**Background:**

Sit-to-stand movements are a necessary part of daily life, and excessive mechanical stress on the articular cartilage has been reported to encourage the progression of osteoarthritis. Although a change in hip joint angle at seat-off may affect hip joint contact force during a sit-to-stand movement, the effect is unclear. This study aimed to examine the effect of the hip joint angle at seat-off on the hip joint contact force during a sit-to-stand movement by using a computer simulation.

**Methods:**

A musculoskeletal model was created for the computer simulation, and eight muscles were attached to each lower limb. Various sit-to-stand movements were generated using parameters (e.g., seat height and time from seat-off to standing posture) reported by previous studies. The hip joint contact force for each sit-to-stand movement was calculated. Furthermore, the effect of the hip joint angle at seat-off on the hip joint contact force during the sit-to-stand movement was examined. In this study, as the changes to the musculoskeletal model parameters affect the hip joint contact force, a sensitivity analysis was conducted.

**Results and conclusions:**

The hip joint contact force during the sit-to-stand movement increased approximately linearly as the hip flexion angle at the seat-off increased. Moreover, the normal sit-to-stand movement and the sit-to-stand movement yielding a minimum hip joint contact force were approximately equivalent. The effect of the changes to the musculoskeletal model parameters on the main findings of this study was minimal. Thus, the main findings are robust and may help prevent the progression of hip osteoarthritis by decreasing mechanical stress, which will be explored in future studies.

**Electronic supplementary material:**

The online version of this article (10.1186/s12938-018-0610-5) contains supplementary material, which is available to authorized users.

## Introduction

Mechanical stress on the articular cartilage encourages osteoarthritis (OA) progression [1–3]. Hip OA decreases an individual’s range of motion [4–6], muscle strength [6–9], and physical functioning [10]; degrades their health-related quality of life [11]; and results in hip joint pain [5, 6, 12, 13]. Therefore, the reduction in the mechanical stress on the hip articular cartilage is important in daily life to prevent OA morbidity and progression.

According to a previous study [14], individuals must engage in sit-to-stand (STS) movements daily, with typically an approximate of 60 movements per day. The STS movement requires the activation of the hip extension muscle [15, 16], hip extension moment [16–21], and hip extensor muscle forces [22]. The hip joint contact force (i.e., the actual force on a hip joint surface including the effect of muscle activity) increases with the increased muscular contraction across the hip joint. Therefore, it is important to clarify the STS movement that reduces the hip extensor muscle forces and hip joint contact force.

In a typical STS movement from a chair, the buttocks lose contact with the chair, following hip flexion and a forward inclination of the trunk [23]. The sum of the head and trunk masses accounts for 56% of the body mass [24], and change in the hip flexion angle approximately corresponds to the change in the center of the mass of the trunk. Yoshioka et al. [18] reported that the peak hip extension moment during an STS movement with the large hip flexion angle at seat-off is large. Furthermore, Doorenbosch et al. [16] examined the joint moments and muscle activations of two types of STS movements: the natural STS transfer and the STS transfer with full trunk flexion. However, they did not examine the hip joint contact force during these movements [16, 18]. In contrast, several studies [25–27] have directly measured the hip joint contact force during an STS movement. However, the relationship between the hip joint angle at seat-off and hip joint contact force during an STS movement was not evaluated [25–27]. As such, no study has examined the effect of the hip joint angle at seat-off on the hip joint contact force during an STS movement, although the effect may help prevent hip OA progression.

Therefore, the present study aimed to examine the effect of the hip joint angle at seat-off on the hip joint contact force during an STS movement. Although this task cannot be accomplished by using only an experiment, it is necessary to thoroughly generate various STS movements to clarify STS movement patterns with minimum and maximum hip joint contact forces. Thus, we generated various STS movements by using a computer simulation to examine this effect. We then hypothesized that the hip joint contact force during an STS movement increases with the increased hip flexion angle at seat-off.

## Methods

### Computer simulation

#### Musculoskeletal model

A two-dimensional musculoskeletal model composed of the head–arms–trunk (HAT), thighs, shanks, and feet was created. The height and weight were set to 1.74 m and 73.8 kg, respectively [18]. The length, mass, center of mass, and gyration radius of each segment were also set accordingly [28, 29]. The horizontal position from the heel to ankle joint was set based on the study by Yoshioka et al. [18]. The musculoskeletal model was assumed symmetrical.

**Fig. 1:**
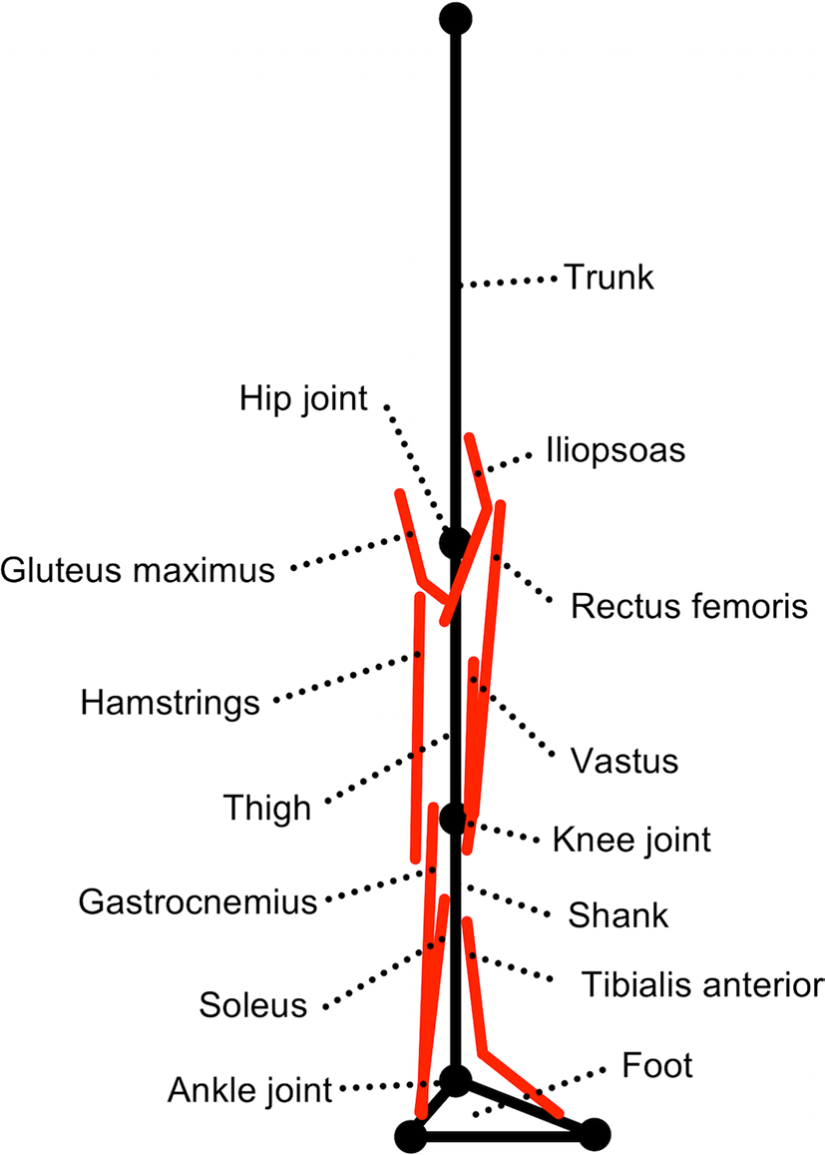
Musculoskeletal model. Eight muscles (iliopsoas, gluteus maximus, vastus, rectus femoris, hamstrings, tibialis anterior, soleus, and gastrocnemius) were attached to the right lower limb. Furthermore, the muscles of the left lower limb were assumed to perform the same activities as those of the right lower limb

Eight muscles (iliopsoas, gluteus maximus, vastus, rectus femoris, hamstrings, tibialis anterior, soleus, and gastrocnemius) were attached to the right lower limb (Fig. 1). The muscles of the left lower limb were assumed to perform the same activities as those of the right lower limb. All muscles comprised contractile and series elastic elements.

We used the force–length curve function [30], and created the force–velocity curve according to that by Zajac [31]. The raw data of the muscle fiber length ratios, except the gluteus maximus, as a function of the hip, knee, and ankle joint angles, were exported from the Full Body Model [32, 33] on OpenSim [32]. Meanwhile, the raw data of the muscle fiber length ratio of the gluteus maximus was exported from the Hip Musculoskeletal Model on OpenSim. These ratios were used in the current study after being approximated using a quadratic function for each muscle. The muscle fiber velocities were calculated using the differentiation muscle fiber length with respect to time and normalized using maximum muscle fiber velocities. The sum of the products of the muscle lever arm and muscle force (contractile element) for each muscle is constraint in this study. Therefore, even though the passive element is considered, a large change in the hip contact force during an STS movement is not expected because of the existence of the constraint. Thus, the parallel elastic element was not considered in this study. We excluded the pennation angle for each muscle because it showed a very low sensitivity [34].

#### STS movement generation

**Fig. 2:**
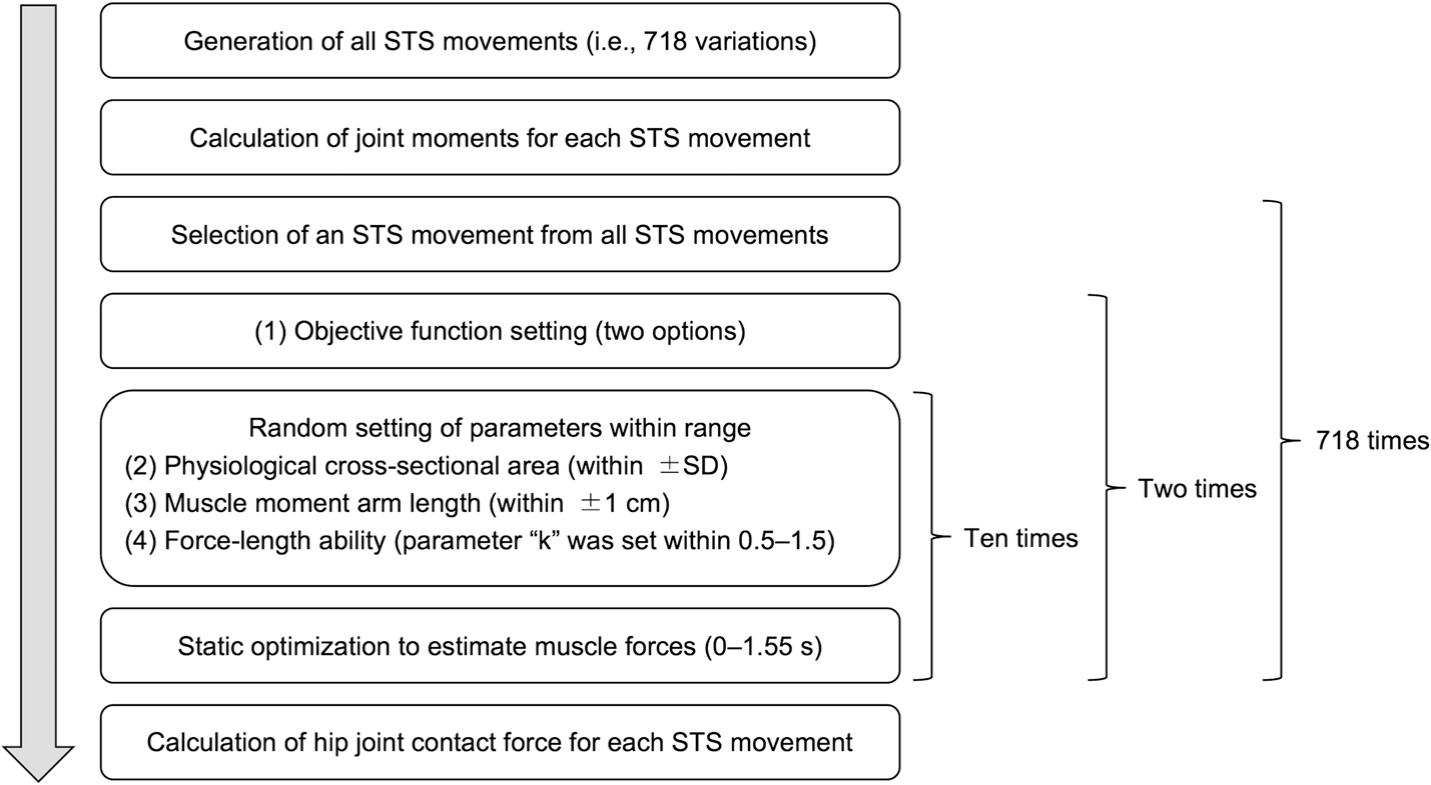
Simulation flowchart. In an STS movement, static optimization is conducted for each frame. The muscle forces during an STS movement are estimated 20 times by using variable parameters. Therefore, estimations of muscle forces were conducted 14,360 (= 20 × 718) times in this computer simulation study

**Fig. 3:**
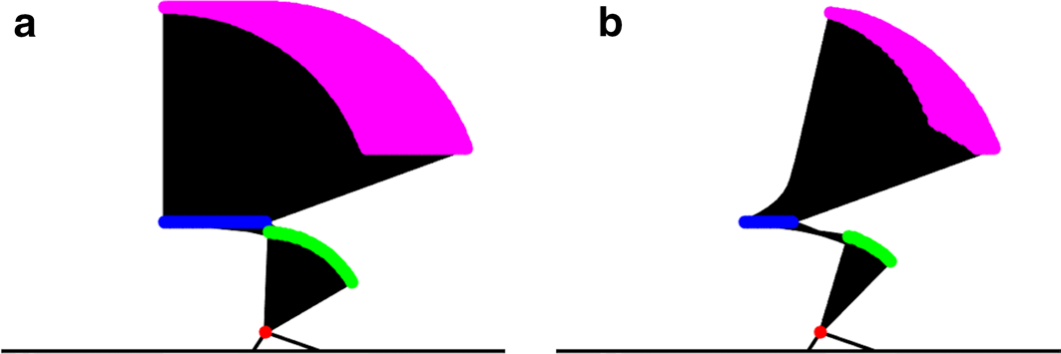
Postures at seat-off. The colors magenta, blue, green, and red indicate the top of the trunk, hip joint, knee joint, and ankle joint, respectively. The hip joint height is set to 0.513 m [18]. **a** All the postures generated at seat-off (2911 variations) and **b** adopted postures at seat-off (718 variations)

Figure 2 shows the computer simulation flowchart. The first step was to generate various postures at seat-off. The *X* and *Y* axes correspond to the horizontal (right direction = positive) and vertical (upward direction = positive) axes, respectively. The hip joint height (*Y* component) at seat-off was set to 0.513 m [18], while the *X* component of the hip joint was set posterior to the ankle joint in the 0–40 cm range (1 cm increments (i.e., 41 variations)). The trunk position was set to range from a point perpendicular to the floor to a position 70° clockwise from that point (i.e., 71 variations). Thus, 2911 postures at seat-off (41 × 71 variations) were generated (Fig. 3a).

**Fig. 4:**
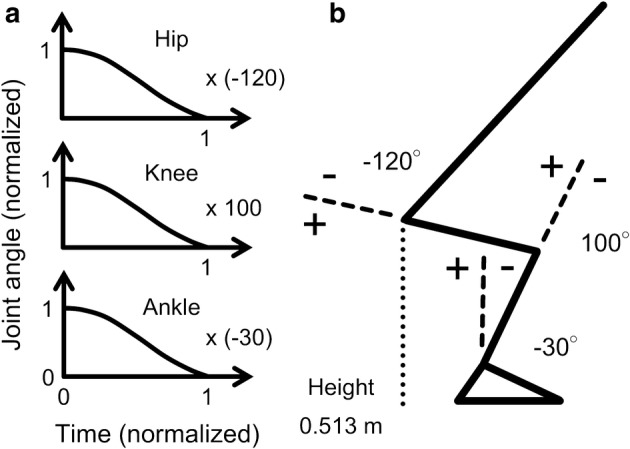
Illustration of **a** normalized joint angles and **b** a posture at seat-off. The normalized joint angles of the hip, knee, and ankle joints are multiplied by the hip, knee, and ankle angles at seat-off, respectively, to generate an STS movement

The next step is to generate various STS movements by using various postures at seat-off. Yoshioka et al. [18] reported that the changes of the hip, knee, and ankle joint angles from seat-off to the standing posture are nonlinear, and the waveforms resemble a cosine curve (0–π). Therefore, in the present study, cosine curves (0–π) were used as hip, knee, and ankle joint angles during an STS movement, and the waveforms were normalized under the same procedure as that used in the study by Yoshioka et al. [18]. The normalized joint angles of the hip, knee, and ankle joints obtained from cosine curves were multiplied by the hip, knee, and ankle joint angles at seat-off, respectively (Fig. 4a, b). Therefore, 2911 STS movements were generated.

The STS movement was excluded if the center of the body mass moved outside the support base (i.e., foot length) [18]. Yoshioka et al. [18] determined the hip, knee, and ankle joint angles at seat-off ranges of 89–138° flexion, 102–114° flexion, and 19–39° dorsiflexion, respectively. In the present study, the STS movements satisfying the hip, knee, and ankle joints at seat-off ranges of 85–145° flexion, 95–120° flexion, and 15–45° dorsiflexion, respectively, were adopted. The final number of the adopted STS movements was 718. Figure 3b shows the final adopted postures at seat-off of 718 STS movements. The time of the STS movement from the seat-off to standing posture was set to 1.55 s based on a previous study [16]. To exclude the effect of the time required by an STS movement on the hip joint contact force, the constant movement time was used.

#### Calculation of the joint moments, muscle forces, and hip joint contact force

The hip, knee, and ankle joint moments of all the STS movements (i.e., 718 variations) were calculated using inverse dynamics (Additional file 1: Appendices A, B, C Ref. Yoshioka et al. [18] for further detail).

Static optimization [35] was used to estimate the muscle forces. Note that the difference in the objective function affects the muscle force values [22]. Therefore, two objective functions, namely the sum of the muscle activations squared and sum of the muscle stress squared, were used in this study.

The first objective function, which is the sum of the muscle activations squared, is presented as follows:

1 $$J_{1} = \mathop \sum \limits_{m = 1}^{8} \left( {\frac{{F_{CE, m} }}{{F_{MAX, m} }}} \right)^{2} .$$

The second objective function, which is the sum of the muscle stresses squared, is:

2 $$J_{2} = \mathop \sum \limits_{m = 1}^{8} \left( {\frac{{F_{CE, m} }}{{PCSA_{m} }}} \right)^{2} ,$$

We used Eq. (4) for equality constraints (i.e., hip, knee, and ankle joint torques) and Eq. (5) to set the lower and upper limits.

3 $$M_{j} = \mathop \sum \limits_{m = 1}^{8} r_{m, j} F_{CE, m} ,$$

4 $$0 \le F_{CE, m} \le F_{MAX, m} ,$$

5 $$F_{N\_MAX, m} = f_{ce} (\tilde{L}_{m} )f_{v} (\dot{L}_{m} ),$$

6 $$F_{MAX, m} = F_{N\_MAX, m} PCSA_{m} \sigma ,$$

where *m*: number of muscles (1–8); *F*_*CE*_: maximum force of the contractile element; *F*_*MAX*_: maximum muscle force of the contractile element; *PCSA*: physiological cross-sectional area; *M*: joint moment; *j*: joint (hip, knee and ankle); *r*: muscle moment arm length; $F_{{N_{MAX} }}$: normalized maximum muscle force of the contractile element; *f*_*ce*_: normalized muscle (force–length curve); *f*_*v*_: normalized muscle (force–velocity curve); $\tilde{L}$: muscle fiber length ratio; $\dot{L}$: normalized muscle fiber length velocity; and *σ*: specific muscle tension.

The physiological cross-sectional areas (PCSAs) for each muscle were set according to the study of Handsfield et al. [36] (Table 1). The specific muscle tension was set to 60 N/cm^2^ [33]. The mean values of the hip, knee, and ankle joint angles at seat-off in this computer simulation were 120° flexion, 109° flexion, and 35° dorsiflexion, respectively. Thus, the constant values of the muscle moment arm lengths at these joint angles were used (Table 1). The moment arm lengths of the gluteus maximus, quadriceps (i.e., patellar tendon), and other muscles were obtained using the Hip Musculoskeletal Model (only flexion and extension components) on OpenSim, the function reported by Herzog et al. [37], and the Full Body Model [32, 33] (only flexion and extension components) on OpenSim, respectively.

**Table 1:** Muscle parameters in this study

	Physiological cross-sectional area (cm^2^)		Muscle moment arm length (m)		
	Mean	SD	Hip	Knee	Ankle
Iliopsoas	28.9	6.9	0.022	–	–
Gluteus maximus	46.8	8.7	0.027	–	–
Vastus	157.4	31.5	–	0.044	–
Rectus femoris	34.8	7.4	0.014	0.044	–
Hamstrings	73.0	16.3	0.011	0.028	–
Tibialis anterior	15.8	2.9	–	–	0.043
Soleus	124.1	24.9	–	–	0.026
Gastrocnemius	73.1	16.0	–	0.022	0.031

The hip joint contact forces for each STS movement were calculated using the hip joint force and muscle forces across the hip joint. The muscle lines of action were determined according to the study by Hoy et al. [38], while the ischial tuberosity was set based on the gluteus maximus via point (Additional file 1: Appendices A, B, C). The hip joint contact forces for each STS movement were calculated using the following equation:

7 $$HJCF = \left\| {\tilde{v}_{HJF} - \sum\limits_{n = 1}^{4} {F_{CE,n} \tilde{e}_{n} } } \right\|,$$

where *HJCF*: hip joint contract function; $\tilde{v}_{HJF}$: hip joint force vector; *n*: number of muscles across the hip joint (1–4); *F*_*CE*_: muscle force of the contractile element; and $\tilde{e}_{n}$: unit vector of the muscle line of action.

Doorenbosch et al. [16] reported that the hip flexion angle at the seat-off was 93.4 ± 8.4° flexion. Therefore, the STS movements, at which the hip joint angle at seat-off satisfied the 93 ± 8° flexion, were regarded as the normal STS movements, which were 110 in total.

#### Sensitivity analysis

The musculoskeletal model parameters affect the muscle force values [34]. In a previous study [22] on STS movements, the objective function PCSA, muscle moment arm length, and force–length ability were adjusted to conduct a sensitivity analysis. In this study, a sensitivity analysis was conducted by changing four parameters: objective function, PCSA, muscle moment arm length, and force–length ability.

Two objective functions, namely the sum of the muscle activations squared and sum of the muscle stress squared, were used. Random values within the range of ± standard deviation of the PCSAs were added to the mean values of the PCSAs [36] (Table 1). Arnold et al. [39] showed a possible occurrence of a ~ 2 cm difference in the muscle moment arm length. Therefore, random values within ± 1 cm for each muscle were added to the mean values of the muscle moment arm length. The tendon slack length is a highly sensitive parameter [40, 41], and its variation affects the change of the normalized maximum muscle force. Hence, in this study, the normalized maximum muscle forces for each muscle were changed using parameter *k* (Additional file 1: Appendices A, B, C). Scovil and Ronsky [42] varied various parameters within ± 50%, consequently generating *k* as a random value within ± 50% to change the normalized maximum muscle forces (i.e., force–length ability). All custom codes for this computer simulation were written in MATLAB (MathWorks, Natick, MA, USA) and Scilab (Scilab Enterprises, Versailles, France).

## Results

**Fig. 5:**
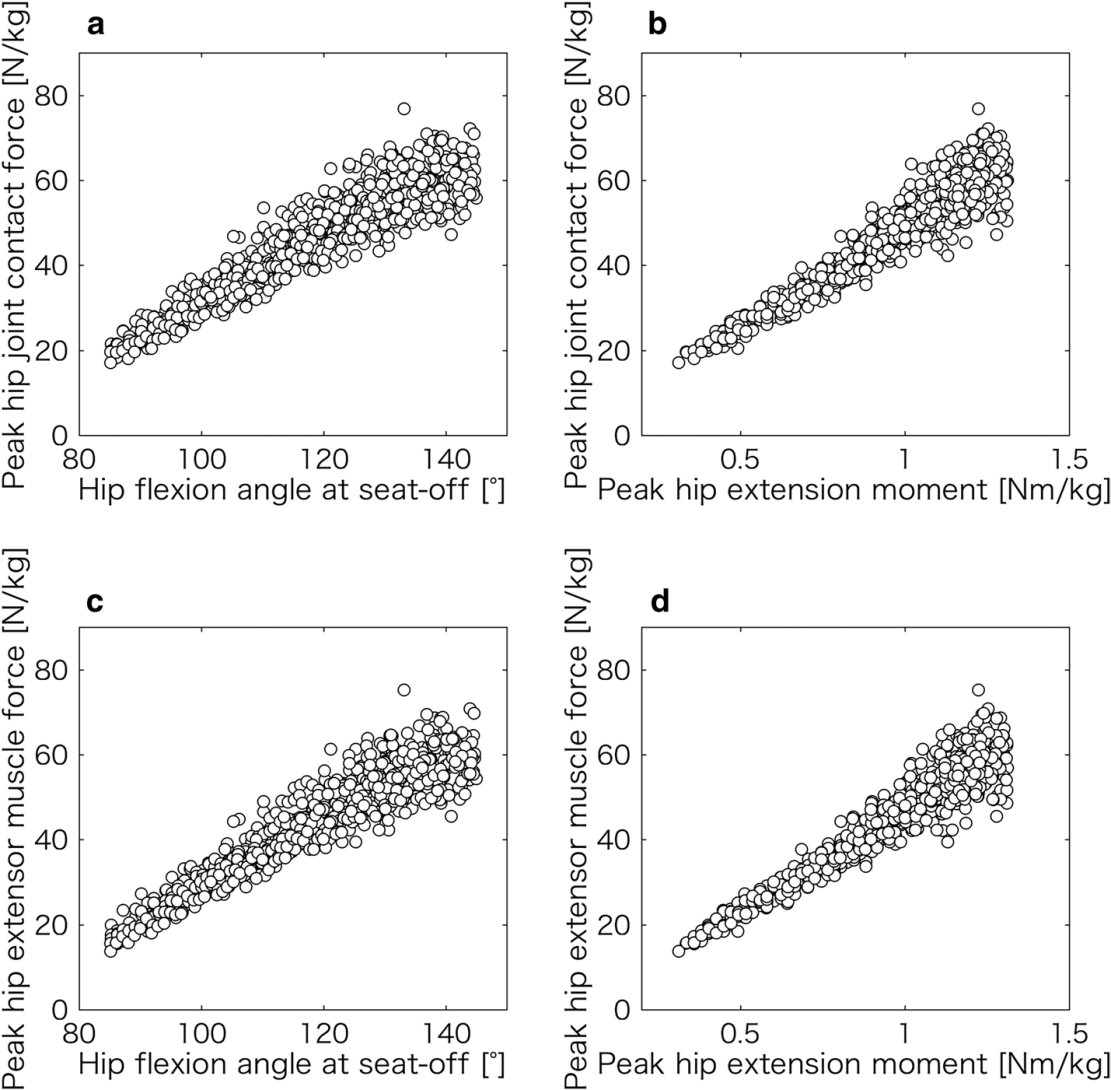
Relationship among the hip joint angle at seat-off, peak hip joint contact force, peak hip extensor muscle force, and peak hip extension moment during the STS movements. All relationships in **a**–**d** are approximately linear

Figure 5a–d show the relationship among the hip joint angles at seat-off, peak hip joint contact force, peak hip extensor muscle force (i.e., sum of the peak muscle forces of the gluteus maximus and hamstrings), and peak hip extension moment during the STS movements. The influence of the parameter (i.e., objective function, PCSA, muscle moment arm length, and force–length ability) variation on the results in Fig. 5 was small, and Fig. 5a–d indicate that these relationships were approximately linear.

**Fig. 6:**
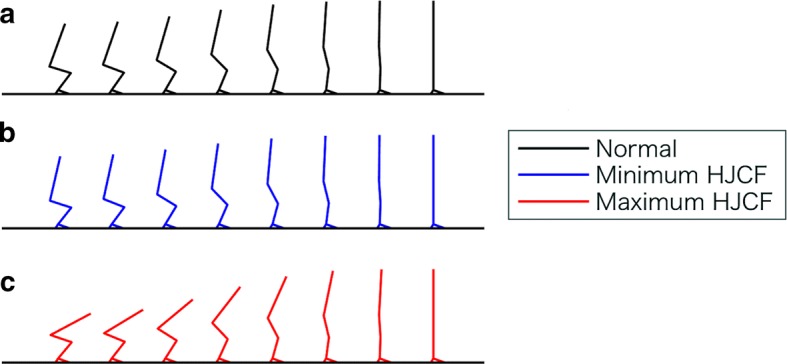
STS movements. **a** Normal STS movement. Doorenbosch et al. [16] reported that the hip joint angle at seat-off is 93.4 ± 8.4°. Thus, the STS movement when the hip flexion angle at seat-off is closest to 93° is regarded as a normal STS movement (black solid line). **b**, **c** STS movement when the hip joint contact forces are minimum (blue solid line) and maximum (red solid line), respectively

Figure 6 shows the STS movement patterns, where Fig. 6a–c show the normal STS movement, STS movement yielding the minimum peak hip joint contact force, and STS movement yielding the maximum peak hip joint contact force, respectively.

Table 2 lists the results of the present and previous studies. The sum of the peak hip and peak knee joint moments, joint moments (static component) of the hip, knee, and ankle joints when the absolute value is a minimum, mean of the peak muscle forces, and mean of the peak hip joint contact force were specifically compared in this study with those reported in the previous studies.

**Table 2:** Comparison of the results of this study and of the previous studies

	This study	Previous studies
Joint moments (Nm/kg)		
Minimum |Hip extension moment|		
Peak hip extension moment	0.24*	0.24^a^
Minimum |Knee extension moment|		
Peak knee extension moment	0.47*	0.51^a^
Minimum |Ankle plantarflexion moment|		
Peak ankle plantarflexion moment	− 0.07*	0.02^a^
All STS movements (718 variations) Sum of the peak hip and peak knee joint moments	1.51*	1.53^a^
Normal STS movements (110 variations) Peak hip extension moment	0.52 (0.10)	0.71 (0.16)^b^ 0.62 (0.12)^c^
Muscle forces (mean peak) (N/kg)		
All STS movements (718 variations)		
Gluteus maximus	19.0^†^	5.6^d^
Hamstrings	25.8^†^	7.8^d^
Quadriceps (vastus and rectus femoris)	37.6^†^	36.5^d^
Sum of the peak hip and peak knee extensor muscle forces	82.4^†^	44.7^d^
Normal STS movements (110 variations)		
Gluteus maximus	16.9^‡^	–
Hamstrings	7.0^‡^	–
Quadriceps (vastus and rectus femoris)	32.4^‡^	–
Sum of the peak hip and peak knee extensor muscle forces	56.3^‡^	–
Joint contact force (N/kg)		
Normal STS movements (110 variations)		
Mean of the peak hip joint contact force	26.3^‡^	19.2^e^
		21.3^f^
		19.6^g^

^a^Yoshioka et al. [18]. The seat height was 0.4 m

^b^Inai et al. [43]. The seat height was 0.4 m

^c^Doorenbosch et al. [16]. The seat height was unknown

^d^Yoshioka et al. [22]. The seat height was 0.4 m

^e^Bergmann et al. [25]. The seat height was 0.5 m. The value was calculated from {(1.81 BW + 2.08 BW + 1.82 BW + 2.20 BW + 1.90 BW)/5} × 9.8 m/s^2^. BW: body weight

^f^Bergmann et al. [27]. The seat height was 0.45 m. The value was calculated from 1600 N/75 kg

^g^Stansfield et al. [26]. The seat height was unknown. The value was calculated from 2.0 BW × 9.8 m/s^2^

* The static components of the joint moments in this study were compared with the values reported by a previous study because the STS movements reported by Yoshioka et al. [18] were slow (4.12–10.98 s)

^†^The mean value was calculated from the adopted STS movements of 878 variations because Yoshioka et al. [22] calculated their mean value from various STS movements of 160,086 variations

^‡^The seat height in this study was 0.4 m. The mean value was calculated from the normal STS movements (i.e., the range of the hip flexion angle at seat-off was 93 ± 8°)

From the 7180 solutions, optimal solutions (i.e., muscle forces) were found 6617 times when the first objective function (Eq. 1) was used for optimization. Meanwhile, optimal solutions were found 5850 times when the second objective function (Eq. 2) was used for optimization. The estimated muscle forces exceeded the upper limit (i.e., maximum muscle force of the contractile element; Eq. 4) when optimal solutions were not found.

## Discussions

### Main findings

The main findings of this study are as follows:

1. The peak hip joint contact force during the STS movement increases with the hip flexion angle at seat-off (Fig. 5a).
2. The normal STS movement and STS movement yielding a minimum hip joint contact force are approximately equivalent (Fig. 6a, b).

Although the previous studies [16, 18] examined the joint moments during the STS movements from different hip joint angles at seat-off, the hip joint contact force during the STS movement was not investigated. Several studies [25–27] directly measured the hip joint contact force. However, the relationship between the hip flexion angle at seat-off and the hip joint contact force during the STS movement has not yet been examined. Therefore, the relationship evaluated in this study constitutes a novel research question.

### Validity and sensitivity analysis for the STS movements

In this computer simulation study, 718 STS movements were adopted. The number was smaller than that reported by Yoshioka et al. [18] (i.e., 160,086 STS movements). We generated the various STS movements using a different method than the method used by them [18]; however, the comparisons in Table 2 indicate that the values of the joint moments in this study were approximately identical to those determined by Yoshioka et al. [18]. Furthermore, the mean of the peak hip extension moment during normal STS movements (110 variations) in the present computer simulation study is 0.52 (0.10) Nm/kg. According to previous experimental studies, the peak hip extension moments during normal STS movements were 0.71 (0.16) [43] and 0.62 (0.12) Nm/kg [16]. Hence, the joint moment values calculated in this study are thought to be quantitatively reasonable.

The peak muscle forces of the gluteus maximus and hamstrings during the STS movement in this study were larger than those reported by Yoshioka et al. [22] (Table 2). In addition, the muscle moment arm lengths in this study were shorter than those reported by Yoshioka et al. [22]. Hence, we tested the computer simulation by using the muscle moment arm lengths reported by Yoshioka et al. [22]. As a result (Additional file 1: Appendices A, B, C), the peak muscle forces of the gluteus maximum and hamstrings during the STS movements in the computer simulation were 5.5 and 9.7 N/kg, respectively. The peak muscle forces of the gluteus maximum and hamstrings during the STS movements in the previous study [22] were 5.6 and 7.8 N/kg, respectively. Therefore, the difference between this study and that of Yoshioka et al. [22] is considered to be due to the difference of the muscle moment arm length. Although a number of studies [22, 44–48] also estimated muscle forces during STS movements, no study has invasively measured the muscle forces during STS movements. Thus, we could not show whether the muscle forces estimated in this study are quantitatively valid.

Fortunately, some previous studies directly measured the hip joint contact force during STS movements [25–27]. Bergmann et al. [25, 27] reported that the values of the peak hip joint contact forces during the STS movement were 21.3 and 19.2 N/kg for seat heights of 0.45 and 0.5 m, respectively. These values are smaller than 26.3 N/kg calculated in this study (Table 2). However, the seat height here was 0.4 m. Yoshioka et al. [19] reported that the peak hip joint moment significantly increased as the seat height decreased within the 0.6–0.4 m range. The peak hip joint moment during the STS movement from a seat height of 0.4 m was approximately 1.3 times that from a seat height of 0.5 m [19]. Moreover, the relationship between the peak hip extension moment and peak hip joint contact force during the STS movement was approximately linear (Fig. 5b). Therefore, the peak hip joint contact force of 20.2 N/kg (i.e., 26.3 N/kg/1.3 times) in this study was close to 19.2 N/kg reported by Bergmann et al. [25]. In other words, the peak hip joint contact force in this study was reasonable, and the estimated hip extensor muscle forces may be indirectly proper.

We conducted a sensitivity analysis because the musculoskeletal model parameters affected the muscle force values [22, 34]. The objective function, PCSA, muscle moment arm length, and force–length ability were changed. However, the variation in the musculoskeletal model parameters had a small effect on the primary results of this study. Thus, the main findings of this study were robust.

### Clinical application

This study revealed that the peak hip joint contact force during the STS movement increased as the hip flexion angle at seat-off increased. Moreover, the mechanical stress on the articular cartilage encourages OA progression [1–3]. Therefore, the STS movements corresponding to a large hip flexion angle at seat-off may increase the risk of OA progression.

According to previous studies, hip OA decreases an individual’s range of motion in the hip joint in the sagittal plane [4–6, 49]. Therefore, hip OA patients with a reduced range of motion in the hip joint in the sagittal plane cannot perform normal STS movements. Therefore, we believe that it is important to examine the effect of the hip angle in the sagittal plane at seat-off on the hip joint contact force during STS movements. In addition, the main findings of this study may help to prevent to the progression of hip OA by decreasing mechanical stress, which will be explored in future studies.

### Limitation

A two-dimensional sagittal musculoskeletal model can evaluate the pure effect of muscle forces in the sagittal plane on the hip joint contact force; however, the gluteus medius muscle was not considered (i.e., muscle forces in the frontal plane). Burnfield et al. [15] reported that the peak electromyography of the gluteus medius during normal STS movements was only 11% relative to the results of a maximal manual muscle test. Therefore, roughly 4.1 N/kg, which was calculated from (45.6 cm^2^ × 0.11 × 60 N/cm^2^)/73.8 kg, may be added to the hip joint contact force estimated in this study by using the PCSA reported by Handsfield et al. [36]. An analysis using a three-dimensional musculoskeletal model may be necessary to estimate a realistic hip joint contact force during the STS movement. Furthermore, we assumed symmetrical STS movements in the present study. However, elderly and hip OA patients may change STS movement in order to maintain balance and/or reduce hip pain. Therefore, we should examine hip joint contact force during more realistic STS movements in future studies.

## Conclusion

We examined the effect of the hip joint angle at seat-off on the hip joint contact force during the STS movement. The following conclusions were obtained.

1. The peak hip joint contact force during the STS movement increases with the hip flexion angle at seat-off.
2. The normal STS movement and the STS movement yielding a minimum hip joint contact force are approximately equivalent.

## Additional file

**Additional file 1.** Muscle parameters (Appendix A), operation of the normalized maximum muscle forces (Appendix B), and computer simulation using other muscle moment arm lengths (Appendix C).

## Abbreviations

OA: osteoarthritis
STS: sit-to-stand movement

## References

[1] Griffin TM, Guilak F (2005). The role of mechanical loading in the onset and progression of osteoarthritis. Exerc Sport Sci Rev.

[2] Guilak F (2011). Biomechanical factors in osteoarthritis. Best Pract Res Clin Rheumatol.

[3] Lucchinetti E, Adams CS, Horton WE, Torzilli PA (2002). Cartilage viability after repetitive loading: a preliminary report. Osteoarthr Cartil.

[4] Steultjens MP, Dekker J, van Baar ME, Oostendorp RA, Bijlsma JW (2000). Range of joint motion and disability in patients with osteoarthritis of the knee or hip. Rheumatology.

[5] Holla JFM, Steultjens MPM, van der Leeden M, Roorda LD, Bierma-Zeinstra SMA, den Broeder AA (2011). Determinants of range of joint motion in patients with early symptomatic osteoarthritis of the hip and/or knee: an exploratory study in the CHECK cohort. Osteoarthr Cartil.

[6] van Baar ME, Dekker J, Lemmens JA, Oostendorp RA, Bijlsma JW (1998). Pain and disability in patients with osteoarthritis of hip or knee: the relationship with articular, kinesiological, and psychological characteristics. J Rheumatol.

[7] Arokoski MH, Arokoski JPA, Haara M, Kankaanpää M, Vesterinen M, Niemitukia LH (2002). Hip muscle strength and muscle cross sectional area in men with and without hip osteoarthritis. J Rheumatol.

[8] Rasch A, Byström AH, Dalen N, Berg HE (2007). Reduced muscle radiological density, cross-sectional area, and strength of major hip and knee muscles in 22 patients with hip osteoarthritis. Acta Orthop.

[9] Loureiro A, Mills PM, Barrett RS (2013). Muscle weakness in hip osteoarthritis: a systematic review. Arthritis Care Res.

[10] Lin YC, Davey RC, Cochrane T (2001). Tests for physical function of the elderly with knee and hip osteoarthritis. Scand J Med Sci Sports.

[11] Salaffi F, Carotti M, Stancati A, Grassi W (2005). Health-related quality of life in older adults with symptomatic hip and knee osteoarthritis: a comparison with matched healthy controls. Aging Clin Exp Res.

[12] Tateuchi H, Koyama Y, Tsukagoshi R, Kuroda Y, So K, Goto K (2016). Associations of radiographic degeneration and pain with daily cumulative hip loading in patients with secondary hip osteoarthritis. J Orthop Res.

[13] Tateuchi H, Akiyama H, Goto K, So K, Kuroda Y, Ichihashi N (2018). Sagittal alignment and mobility of the thoracolumbar spine are associated with radiographic progression of secondary hip osteoarthritis. Osteoarthr Cartil.

[14] Dall PM, Kerr A (2010). Frequency of the sit to stand task: an observational study of free-living adults. Appl Ergon.

[15] Burnfield JM, Shu Y, Buster TW, Taylor AP, McBride MM, Krause ME (2012). Kinematic and electromyographic analyses of normal and device-assisted sit-to-stand transfers. Gait Posture.

[16] Doorenbosch CA, Harlaar J, Roebroeck ME, Lankhorst GJ (1994). Two strategies of transferring from sit-to-stand; the activation of monoarticular and biarticular muscles. J Biomech.

[17] Eitzen I, Fernandes L, Nordsletten L, Snyder-Mackler L, Risberg MA (2014). Weight-bearing asymmetries during Sit-To-Stand in patients with mild-to-moderate hip osteoarthritis. Gait Posture.

[18] Yoshioka S, Nagano A, Himeno R, Fukashiro S (2007). Computation of the kinematics and the minimum peak joint moments of sit-to-stand movements. Biomed Eng Online.

[19] Yoshioka S, Nagano A, Hay DC, Fukashiro S (2014). Peak hip and knee joint moments during a sit-to-stand movement are invariant to the change of seat height within the range of low to normal seat height. Biomed Eng Online.

[20] Galli M, Crivellini M, Sibella F, Montesano A, Bertocco P, Parisio C (2000). Sit-to-stand movement analysis in obese subjects. Int J Obes Relat Metab Disord.

[21] Yoshioka S, Nagano A, Hay DC, Fukashiro S (2009). Biomechanical analysis of the relation between movement time and joint moment development during a sit-to-stand task. Biomed Eng Online.

[22] Yoshioka S, Nagano A, Hay DC, Fukashiro S (2012). The minimum required muscle force for a sit-to-stand task. J Biomech.

[23] Nuzik S, Lamb R, VanSant A, Hirt S (1986). Sit-to-stand movement pattern. A kinematic study. Phys Ther.

[24] Dempster WT, Gaughran GRL (1967). Properties of body segments based on size and weight. Am J Anat.

[25] Bergmann G, Deuretzbacher G, Heller M, Graichen F, Rohlmann A, Strauss J (2001). Hip contact forces and gait patterns from routine activities. J Biomech.

[26] Stansfield BW, Nicol AC, Paul JP, Kelly IG, Graichen F, Bergmann G (2003). Direct comparison of calculated hip joint contact forces with those measured using instrumented implants. An evaluation of a three-dimensional mathematical model of the lower limb. J Biomech.

[27] Bergmann G, Bender A, Dymke J, Duda G, Damm P (2016). Standardized loads acting in hip implants. PLoS ONE.

[28] Contini R (1972). Body segment parameters: Part II. Artif Limbs.

[29] de Leva P (1996). Adjustments to Zatsiorsky-Seluyanov’s segment inertia parameters. J Biomech.

[30] Brown IE, Cheng EJ, Loeb GE (1999). Measured and modeled properties of mammalian skeletal muscle. II. The effects of stimulus frequency on force–length and force–velocity relationships. J Muscle Res Cell Motil.

[31] Zajac FE (1989). Muscle and tendon: properties, models, scaling, and application to biomechanics and motor control. Crit Rev Biomed Eng.

[32] Delp SL, Anderson FC, Arnold AS, Loan P, Habib A, John CT (2007). OpenSim: Open-source software to create and analyze dynamic simulations of movement. IEEE Trans Biomed Eng.

[33] Rajagopal A, Dembia CL, DeMers MS, Delp DD, Hicks JL, Delp SL (2016). Full-Body Musculoskeletal Model for Muscle-Driven Simulation of Human Gait. IEEE Trans Biomed Eng.

[34] Carbone V, van der Krogt MM, Koopman HFJM, Verdonschot N (2016). Sensitivity of subject-specific models to Hill muscle–tendon model parameters in simulations of gait. J Biomech.

[35] Erdemir A, McLean S, Herzog W, van den Bogert AJ (2007). Model-based estimation of muscle forces exerted during movements. Clin Biomech.

[36] Handsfield GG, Meyer CH, Hart JM, Abel MF, Blemker SS (2014). Relationships of 35 lower limb muscles to height and body mass quantified using MRI. J Biomech.

[37] Herzog W, Read LJ (1993). Lines of action and moment arms of the major force-carrying structures crossing the human knee joint. J Anat.

[38] Hoy MG, Zajac FE, Gordon ME (1990). A musculoskeletal model of the human lower extremity: the effect of muscle, tendon, and moment arm on the moment-angle relationship of musculotendon actuators at the hip, knee, and ankle. J Biomech.

[39] Arnold EM, Ward SR, Lieber RL, Delp SL (2010). A model of the lower limb for analysis of human movement. Ann Biomed Eng.

[40] Ackland DC, Lin Y-C, Pandy MG (2012). Sensitivity of model predictions of muscle function to changes in moment arms and muscle–tendon properties: a Monte-Carlo analysis. J Biomech.

[41] Redl C, Gfoehler M, Pandy MG (2007). Sensitivity of muscle force estimates to variations in muscle–tendon properties. Hum Mov Sci.

[42] Scovil CY, Ronsky JL (2006). Sensitivity of a Hill-based muscle model to perturbations in model parameters. J Biomech.

[43] Inai T, Takabayashi T, Edama M, Kubo M (2018). Relationship between movement time and hip moment impulse in the sagittal plane during sit-to-stand movement: a combined experimental and computer simulation study. Biomed Eng Online.

[44] Ellis MI, Seedhom BB, Wright V (1984). Forces in the knee joint whilst rising from a seated position. J Biomed Eng.

[45] Shelburne KB, Pandy MG (2002). A dynamic model of the knee and lower limb for simulating rising movements. Comput Methods Biomech Biomed Eng.

[46] Jo YN, Kang MJ, Yoo HH (2014). Estimation of muscle and joint forces in the human lower extremity during rising motion from a seated position. J Mech Sci Technol.

[47] Caruthers EJ, Thompson JA, Chaudhari AMW, Schmitt LC, Best TM, Saul KR (2016). Muscle forces and their contributions to vertical and horizontal acceleration of the center of mass during sit-to-stand transfer in young, healthy adults. J Appl Biomech.

[48] Bobbert MF, Kistemaker DA, Vaz MA, Ackermann M (2016). Searching for strategies to reduce the mechanical demands of the sit-to-stand task with a muscle-actuated optimal control model. Clin Biomech.

[49] Pua YH, Wrigley TV, Cowan SM, Bennell KL (2009). Hip flexion range of motion and physical function in hip osteoarthritis: mediating effects of hip extensor strength and pain. Arthritis Care Res.

