# Supplementary material for: Effect of hip joint angle at seat-off on hip joint contact force during sit-to-stand movement: a computer simulation study
Source: Biomed Eng Online. 2018 Nov 29;17:177. doi: 10.1186/s12938-018-0610-5 (PMC6267796; doi:10.1186/s12938-018-0610-5)
Supplement: Supplementary file 1 — Additional file 1. Muscle parameters (Appendix A), operation of the normalized maximum muscle forces (Appendix B), and computer simulation using other muscle moment arm lengths (Appendix C). [file 12938_2018_610_MOESM1_ESM.docx]

**Additional file 1: Appendix A**

| Table S1. Coordinate points of eight muscles according to a previous study [1]. | | | | |
| --- | --- | --- | --- | --- |
| Muscle | Point | x [m] | y [m] | Reference frame |
| Iliopsoas* | Origin | 0.0075 | 0.1350 | Trunk |
|  | Via point | 0.0260 | 0.0293 | Trunk |
|  | Insertion | -0.0180 | 0.3351 | Thigh |
| Gluteus maximus* | Origin | -0.0578 | 0.0754 | Trunk |
|  | Via point | -0.0409 | -0.0455 | Trunk |
|  | Insertion | -0.0158 | 0.3519 | Thigh |
| Vastus | Origin | 0.0106 | 0.2026 | Thigh |
|  | Via point | -0.0005 | 0.4056 | Shank |
|  | Insertion | 0.0000 | 0.3700 | Shank |
| Rectus femoris* | Origin | 0.0326 | 0.0323 | Trunk |
|  | Via point | 0.0041 | 0.4084 | Shank |
|  | Insertion | 0.0000 | 0.3700 | Shank |
| Hamstrings* | Origin | -0.0409 | -0.0455 | Trunk |
|  | Insertion | -0.0508 | 0.3321 | Shank |
| Tibialis anterior | Origin | -0.0155 | 0.2175 | Shank |
|  | Via point | 0.0259 | 0.0257 | Shank |
|  | Insertion | 0.1850 | -0.0510 | Foot |
| Soleus | Origin | -0.0292 | 0.2467 | Shank |
|  | Insertion | -0.0365 | -0.0288 | Foot |
| Gastrocnemius | Origin | -0.0203 | 0.0071 | Thigh |
|  | Insertion | -0.0368 | -0.0289 | Foot |
| * The muscle lines were used to calculate hip joint contact force during sit-to-stand (STS) movements. | | | | |

**Additional file 1: Appendix B**

*Operation of the normalized maximum muscle forces*

The change in the normalized muscle forces (i.e., $F_{N\_L}$) affects the change in the normalized maximum force (i.e., $F_{N\_MAX}$). The normalized muscle forces were changed using parameter “k” through the following equation:

${F'}_{N\_L}(\tilde{L}_{m}(t))=k_{m}\left( F_{N\_L}(\tilde{L}_{m}(t))-1 \right)+1$*,*

where:

$${F'}_{N\_L}: changed and normalized muscle force;$$

$$t: time;$$

$$k: random value \left( within 0.5-1.5 \right);and$$

$$F_{N\_L}: normalized muscle force.$$

$f_{ce}$ in Eq. (5) was replaced by ${F'}_{N\_L}$ at the time step, such that a sensitivity analysis can be conducted.


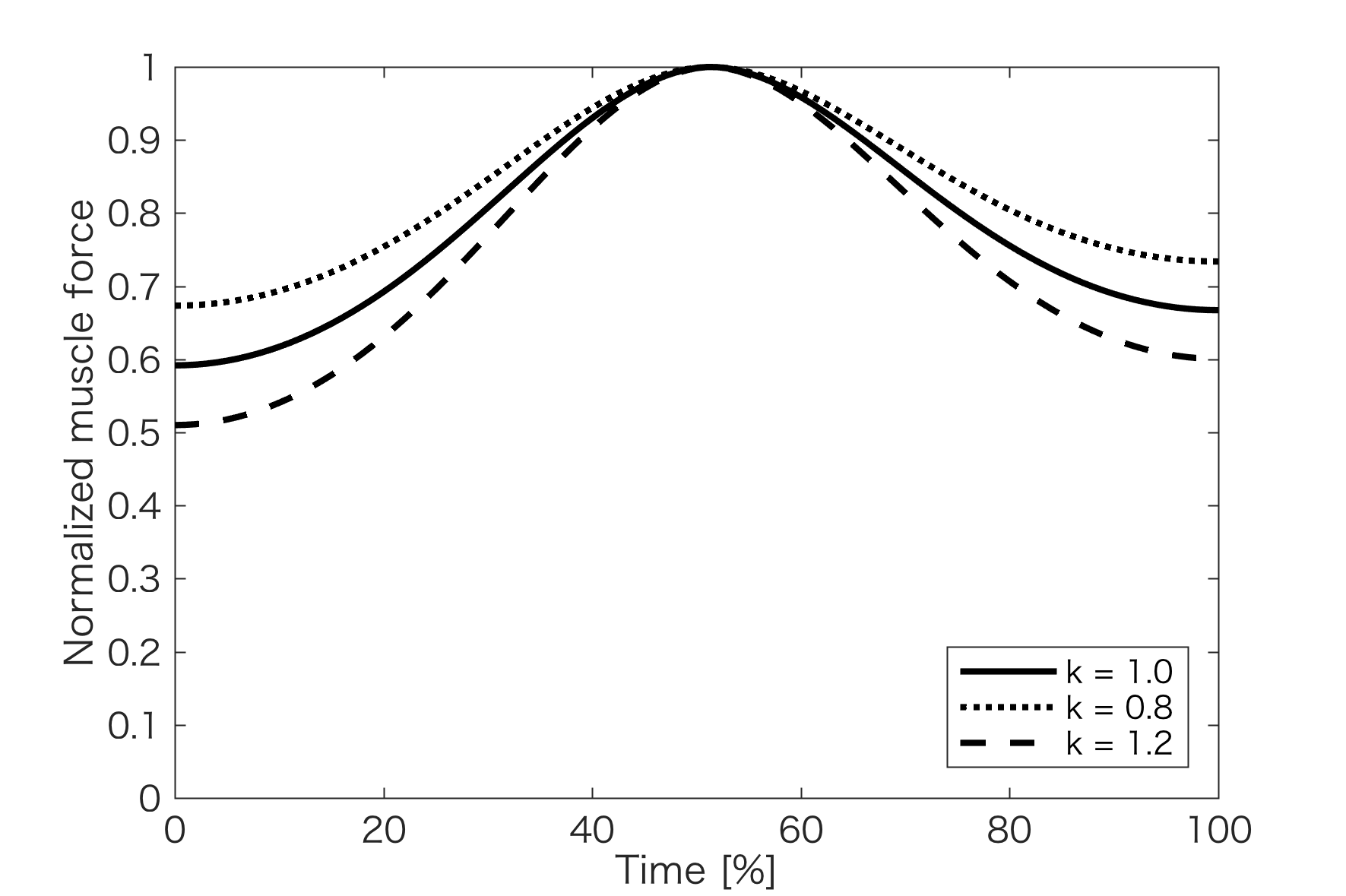


**Figure S1.** Typical examples of the changes in the normalized muscle force

The normalized muscle force is affected by the changing parameter “k.”

**Additional file 1: Appendix C**

*Computer simulation using other muscle moment arm lengths*

We tested the computer simulation using the muscle moment arm lengths reported by Yoshioka et al. [2]. The results are shown in the table below.

**Table S2**. Comparison of the peak muscle forces estimated using different muscle moment arm lengths during the STS movements.

|  | A | B | C |
| --- | --- | --- | --- |
| **Muscle forces (N/kg)** |  |  |  |
| Gluteus maximus | 19.0 | 5.5 | 5.6 |
| Hamstrings | 25.8 | 9.7 | 7.8 |
| Sum of the peak hip and peak knee extensor muscle forces | 82.4 | 42.8 | 44.7 |

A: This study uses the muscle moment arm lengths in Table 1.

B: This study uses the muscle moment arm lengths reported by Yoshioka et al. [2].

C: Yoshioka et al. [2].

**References**

1. A musculoskeletal model of the human lower extremity: the effect of muscle, tendon, and moment arm on the moment-angle relationship of musculotendon actuators at the hip, knee, and ankle. J Biomech. 1990;23:157–69.

2. The minimum required muscle force for a sit-to-stand task. J Biomech. 2012;45:699–705.
